# The role of nuclear factor of activated T cells during phorbol myristate acetate-induced cardiac differentiation of mesenchymal stem cells

**DOI:** 10.1186/s13287-016-0348-6

**Published:** 2016-07-12

**Authors:** Hyang-Hee Seo, Chang Youn Lee, Jiyun Lee, Soyeon Lim, Eunhyun Choi, Jong-Chul Park, Seahyoung Lee, Ki-Chul Hwang

**Affiliations:** Brain Korea 21 PLUS Project for Medical Science, Yonsei University, Seoul, South Korea; Department of Integrated Omics for Biomedical Sciences, Yonsei University, Seoul, South Korea; Institute for Bio-medical Convergence, Catholic Kwandong University, Incheon, South Korea; Cellbiocontrol Laboratory, Department of Medical Engineering, Yonsei University College of Medicine, Seoul, South Korea; Department of Biomedical Sciences, College of Medicine, Catholic Kwandong University, Gangneung, Gangwon-do South Korea

**Keywords:** Phorbol myristate acetate, MSC, NFAT, Small molecule, Cardiac differentiation

## Abstract

**Background:**

We previously reported that phorbol 12-myristate 13-acetate (PMA) treatment can induce the cardiac differentiation of mesenchymal stem cells (MSCs). In the present study, we investigated how PMA induces cardiac differentiation of MSCs, focusing on its effect on the transcription factors responsible for increased cardiac marker gene expression.

**Methods:**

Human MSCs (hMSCs) were treated with 1 μM PMA for 9 days. The expression of MSC markers and cardiac markers in the PMA-treated hMSC, as well as the nuclear translocation of transcription factors, nuclear factor of activated T cells (NFAT), and myogenic differentiation 1 (MyoD), was examined. Transcriptional activity of NFAT was examined by utilizing a green fluorescent protein (GFP) vector containing NFAT motif of human interleukin-2 promoter. The effect of PMA on the expression of key cell cycle regulators was examined.

**Results:**

PMA induces the transcriptional activity of NFAT and MyoD, which have been associated with increased expression of cardiac troponin T (cTnT) and myosin heavy chain (MHC), respectively. Our data suggested that protein kinase C (PKC) mediates the effect of PMA on NFAT activation. Furthermore, PMA treatment increased cell-cycle regulator p27^kip1^ expression, suggesting that PMA triggers the cardiac differentiation program in MSCs by regulating key transcription factors and cell cycle regulators.

**Conclusions:**

The results of this study demonstrate the importance of NFAT activation during PMA-induced MSC differentiation and help us to better understand the underlying mechanisms of small molecule-mediated MSC differentiation so that we can develop a strategy for synthesizing novel and improved differentiation-inducing small molecules.

**Electronic supplementary material:**

The online version of this article (doi:10.1186/s13287-016-0348-6) contains supplementary material, which is available to authorized users.

## Introduction

One common stem cell-based therapy involves the in vitro expansion of stem cells to acquire a sufficient number of cells and the re-introduction of these cells into damaged tissues [1]. However, certain tissues, such as the myocardium, require more than just a high number of therapeutic cells. For example, unsynchronized electric coupling between the transplanted cells and cardiomyocytes can increase the proarrhythmic potential [2]; therefore, therapeutic cells resembling native cardiomyocytes would be preferred in terms of their functionality after transplantation compared with undifferentiated stem cells [3]. One method for inducing differentiation of stem cells into a desired lineage is small molecule treatment.

To date, various small molecules with different biological mechanisms have been utilized to induce the cardiac differentiation of stem cells. For example, a specific p38 mitogen-activated protein kinase (MAPK) inhibitor [4] and a bone morphogenetic protein (BMP) inhibitor [5] induced the cardiac differentiation of embryonic stem cells. Furthermore, several studies have demonstrated that a number of small molecule inhibitors of Wnt signaling promoted cardiomyogenesis in stem cells [6–8]. We have also reported that phorbol 12-myristate 13-acetate (PMA), a well-known protein kinase C (PKC) activator, induced the expression of cardiac marker genes such as myosin heavy chain (MHC) and cardiac troponin T (cTnT) in mesenchymal stem cells (MSCs), thus demonstrating its potential as a cardiac differentiation inducer [9]. Nevertheless, that particular previous study lacked detailed explanations of the underlying mechanisms of how PMA induces cardiac differentiation in MSCs.

Since the small molecule-mediated regulation of protein expression is what renders the phenotypic change toward a desired lineage of cells, it is reasonable to hypothesize that PMA activates certain transcription factors that drive the expression of cardiac marker genes in MSCs. Thus, in the present study, we investigated the effect of PMA on the activation of key transcription factors to further clarify the underlying molecular mechanism of PMA treatment during MSC differentiation.

The transcription factor myogenic differentiation 1 (MyoD) is an early marker of myogenic differentiation and is known to regulate the expression of MHC [10]. MyoD has been reported to convert a variety of cells, such as nerve, liver, and fat cells, to skeletal muscle, demonstrating that it is a powerful master regulator of myogenic differentiation [11]. Because PKC has been reported to be required for β-catenin-indepenent activation of MyoD [12], it is possible that PMA-induced activation of PKC subsequently enhances the transcriptional activity of MyoD and results in increased MHC expression in MSCs. Another cardiac marker, cTnT, is reportedly regulated by nuclear factor of activated T cells (NFAT) [13]. Similar to MyoD, the transcriptional activity of NFAT can also be regulated by PKC-mediated phosphorylation [14]. Thus, the reported cardiac differentiation ability of PMA may involve activation of these transcription factors, namely MyoD and NFAT, and we examined such a possibility in the present study. We investigated the effect of PMA on the activation status of these transcription factors and its related mechanisms.

## Materials and methods

### Induction of cardiogenic differentiation

Human MSCs (hMSC; Lonza, Allendale, NJ, USA) were seeded in 60-mm cell culture plates at a density of 2 × 10^5^ cells/plate, and PMA (Sigma-Aldrich Corp., St. Louis, MO, USA) was added to a final concentration of 1 μM. PMA powder was reconstituted in ethanol to make 1 mM PMA stock solution. For treatment, 1 mM PMA stock was 1:1000 diluted in culture medium. hMSCs were cultured in DMEM-low glucose (Thermo Fisher Scientific, Pittsburgh, PA, USA) supplemented with 10 % fetal bovine serum. The medium was replaced every 3 days with PMA treatment for 9 days.

### Immunofluorescence

Cells were cultured in four-well slide chambers. The cells were permeabilized using 0.1 % Triton X-100 for 10 min. Next, the cells were blocked for 1 h in a blocking solution (2 % bovine serum albumin and 10 % horse serum in phosphate-buffered saline) and incubated with cTnT (ab8295; Abcam, Cambridge, MA, USA), β-MHC (ab15; Abcam) and CD90 antibodies (sc-6071; Santa Cruz Biotechnology, Dallas, TX, USA). FITC-conjugated mouse, rabbit, and goat secondary antibodies (Jackson ImmunoResearch Laboratories, West Grove, PA, USA) were used. Immunofluorescence was detected by confocal microscopy (LSM710; Carl Zeiss Microscopy GmbH, Jena, Germany).

### Co-localization image analysis

The degree of co-localization was measured by ZEN 2009 Light Edition software that calculated the percentage of co-localized pixels relative to all pixels obtained. Following Carl Zeiss Micro-imaging, the Pearson’s correlation coefficient R (PCC) value was calculated. The value for PCC can range from –1 to 1, and a value of 1 indicates that the fluorescence patterns of the two molecules are perfectly matched [15].

### Western blot assay

Equal amounts of proteins were separated by SDS-PAGE. After blocking the membrane with 5 % skim milk for 1 h at room temperature, the membranes were incubated with primary antibodies against NFAT (sc-271127), MyoD (sc-760), p-MARCKS (sc-101730), and p27 (sc-528; all Santa Cruz Biotechnology), LaminB2 (sab2702205; Thermo Fisher Scientific), and β-actin (A5316; Sigma-Aldrich Corp.) overnight at 4 °C. The membrane was washed and incubated for 1 h at room temperature with horseradish peroxidase-conjugated secondary antibodies. The bands were detected by an enhanced chemiluminescence (ECL) reagent (Santa Cruz Biotechnology). The band intensities were quantified using NIH Image J version 1.34e software.

### Nuclear and cytoplasmic extraction

For nuclear and cytoplasmic extraction, NE-PER Nuclear and Cytoplasmic Extraction Reagents (Thermo Fisher Scientific) were used according to the user manual provided by the manufacturer.

### Immunoprecipitation

For immunoprecipitation (IP), cells were lysed in IP lysis buffer (Thermo Fisher Scientific) for 30 min on ice. Cell lysates were centrifuged at 10,000 g for 20 min and the supernatant was retained. After protein quantification, the lysates were incubated with calcineurin A antibody (ab3673; Abcam), and the tubes were rotated for 1 h at 4 °C. After 1 h, Protein G Dynabeads (Thermo Fisher Scientific) were added to the lysates and incubated overnight at 4 °C. The Dynabeads-antibody-calcineurin A complexes were centrifuged at 2500 g for 30 min at 4 °C, and the antibody-calcineurin A complexes were eluted with elution buffer, and the eluted proteins were heated for 10 min at 99 °C and subjected to SDS-PAGE electrophoresis.

### Construction of the pAc-NFAT-GFP vector and transfection

To construct a green fluorescent protein (GFP) vector containing the NFAT binding motif of human interleukin-2 (GGAGGAAAAACTGTTTCATACAGAAGGCGT, corresponding to –286/–257 of the human interleukin-2 promoter [16]), two copies of the NFAT binding motif were synthesized and cloned into the pAcGFP1-1 (Clontech, Mountain View, CA, USA) vector using HindIII and BamHI enzymes. The constructed GFP vectors were delivered to the cells using Lipofectamine LTX (Thermo Fisher Scientific). For six-well plates, 500 ng/well of each vector was used.

### RT-PCR

The expression levels of various genes were analyzed by reverse transcription polymerase chain reaction (RT-PCR). Complementary DNA (cDNA) was generated using 500 ng of total RNA with the Promega Reverse Transcription System according to the manufacturer’s instructions. The PCR conditions consisted of denaturing at 94 °C for 3 min, followed by 35 cycles of denaturation at 94 °C for 30 s, annealing at 94 °C for 30 s, and extension at 72 °C for 30 s, before a final extension at 72 °C for 10 min. RT-PCR products were separated by electrophoresis on a 1.2 % agarose gel (Bio-Rad Laboratories, Inc., Hercules, CA, USA). Glyceraldehyde-3-phosphate dehydrogenase (GAPDH) was used as an internal standard.

### Statistical analysis

Quantitative data were expressed as the means ± SD of at least three independent experiments. For statistical analysis, one-way analysis of variance (ANOVA) with Bonferroni correction was performed using OriginPro 8 SR4 software (ver. 8.0951, OriginLab Corporation, Northampton, MA, USA) if there were more than three groups. A *p* value of less than 0.05 was considered statistically significant.

## Result

### PMA-induced cardiac marker expression in hMSCs

**Fig. 1:**
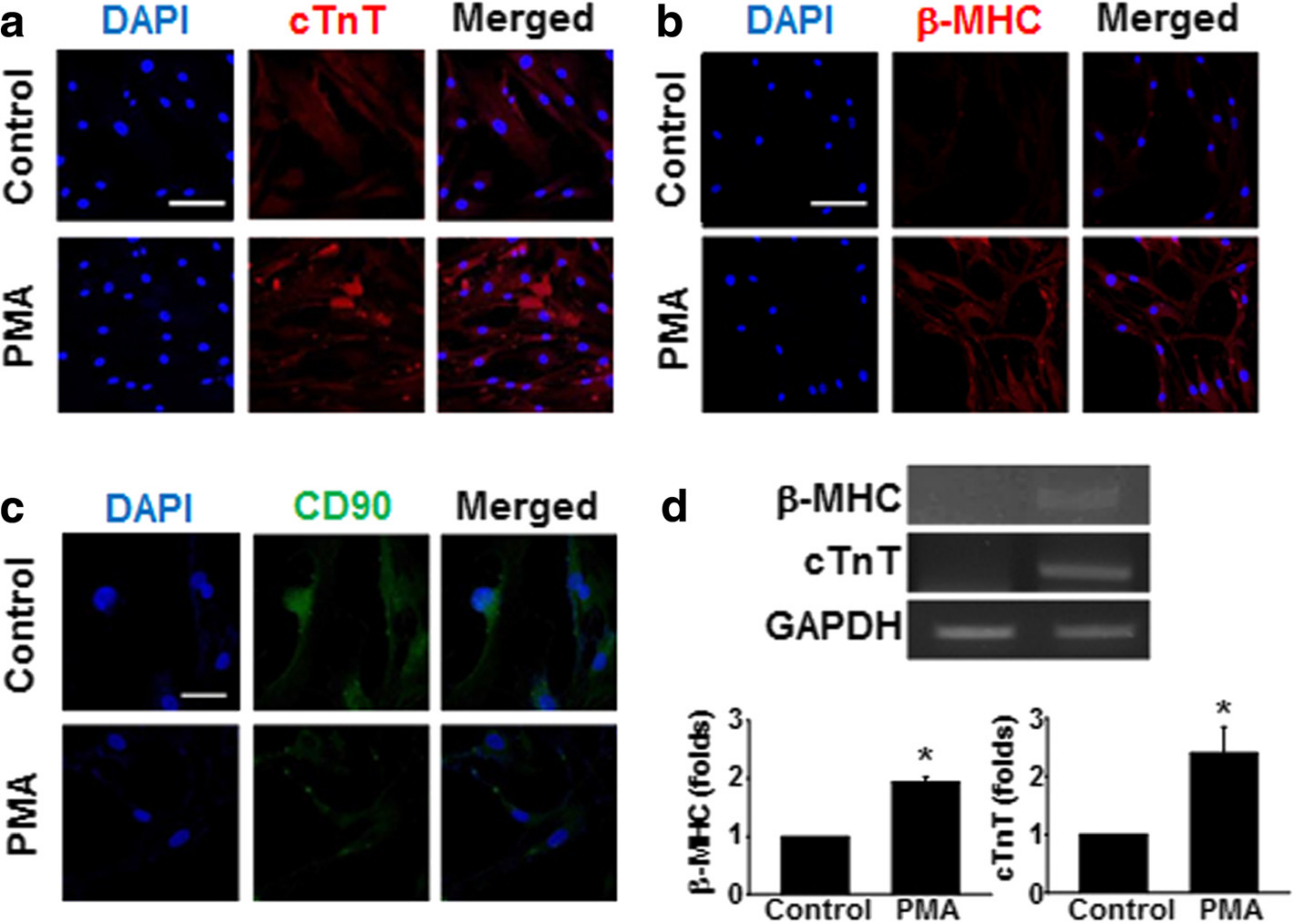
Expression of cardiac and stem cell markers in PMA-treated hMSCs. Immunocytochemical analysis was performed to detect the expression of **a** cTnT (*scale bar* = 100 μm), **b** β-MHC (*scale bar* = 100 μm), and **c** CD90 (*scale bar* = 40 μm) at 9 days after PMA (1 μM) treatment. DAPI was used to stain the nucleus. **d** mRNA expression of β-MHC and cTnT in hMSCs treated with PMA for 9 days was detected by RT-PCR. *p < 0.05, versus control. *cTnT* cardiac troponin T, *MHC* myosin heavy chain, *PMA* phorbol 12-myristate 13-acetate

The cells were treated with PMA (1 μM) for 9 days based on a previous report in which the same regime induced the cardiac differentiation of rMSCs [9]. After 9 days of PMA treatment, the expression levels of the cardiac-specific markers cTnT and MHC increased in the PMA-treated group compared with those of the untreated control (Fig. 1a, b). On the other hand, the expression of the stem cell marker CD90 decreased with PMA treatment compared with that of the untreated control (Fig. 1c). Additionally, the RT-PCR result indicated that PMA increased the expression of both MHC and cTnT at the RNA level compared to control (Fig. 1d). These data suggested that PMA treatment induced the differentiation of hMSCs into cells with cardiomyocyte characteristics.

### PMA induced activation of the transcription factors NFAT and myoD

**Fig. 2:**
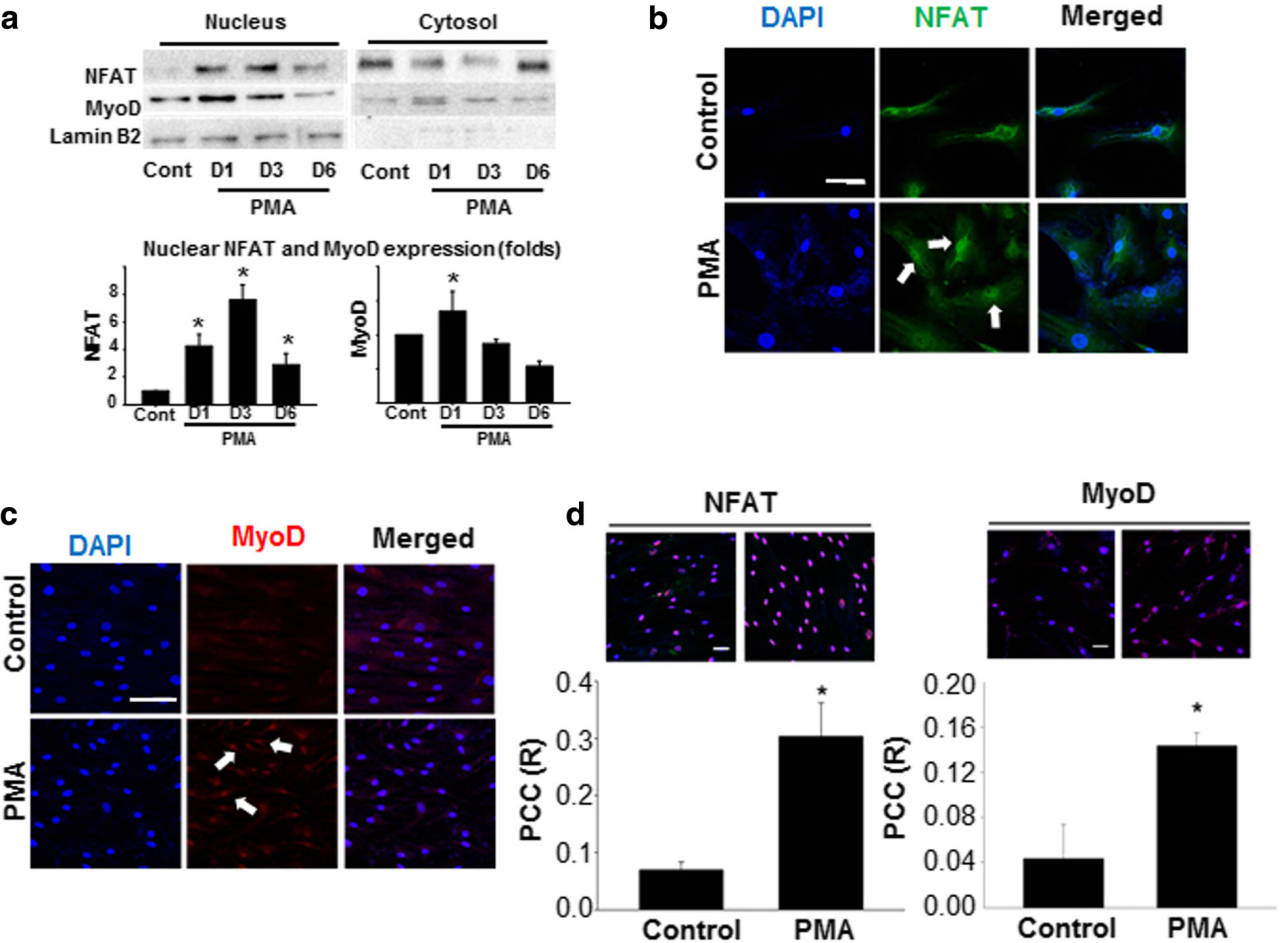
PMA-induced nuclear translocation of MyoD and NFAT in hMSCs. **a** The amount of NFAT and MyoD in the nuclear/cytosolic fraction was examined by Western blot to evaluate the nuclear localization of those transcription factors after PMA (1 μM) treatment. Lamin B2 served as a nuclear control (*Cont*). **b** hMSCs treated with PMA were fixed at day 9 and analyzed by immunofluorescence staining using antibodies against **b** NFAT (*scale bar* = 40 μm) and **c** MyoD (*scale bar* = 100 μm). DAPI was used to stain the nucleus. **d** Calculated co-localization of NFAT and MyoD (shown in *pink*; *scale bar* = 40 μm). *p < 0.05, versus control. *D1* 1 day after PMA treatment, *D3* 3 days after PMA treatment, *D6* 6 days after PMA treatment, *MyoD* myogenic differentiation 1, *NFAT* nuclear factor of activated T cells, *PCC* Pearson’s correlation coefficient, *PMA* phorbol 12-myristate 13-acetate

Nuclear translocation of MyoD and NFAT is a prerequisite for them to have transcriptional activity. Therefore, we examined the subcellular distribution of these transcription factors in hMSCs after PMA treatment. The results of Western blot analysis indicated that the amounts of NFAT and MyoD in the nuclear fraction increased with PMA treatment (Fig. 2a). However, the pattern of the increase was different. The nuclear localization of NFAT gradually increased from day 1 to day 3 and slightly decreased at day 6 in the PMA-treated group. Nevertheless, the amount of nuclear localized NFAT was still higher than that of the control group. On the other hand, the amount of nuclear MyoD increased on day 1, and then decreased thereafter. This suggested that the effect of PMA may have been mediated mainly by NFAT rather than by MyoD.

We performed immunocytochemical staining using antibodies specific to the transcription factors. In untreated control hMSCs, both NFAT and MyoD were evenly distributed without prominent accumulation in the nucleus. However, when the cells were treated with PMA for 3 days, the amount of the transcription factors observed in the nucleus increased noticeably (Fig. 2b, c). To quantify the nuclear localization of both transcription factors, we further conducted a co-localization analysis for the transcription factors and DAPI-stained nuclei. The result indicated that PMA significantly increased the amount of both transcription factors that reside in the nucleus (Fig. 2d). The degree of co-localization was greater for NFAT than MyoD as evidenced by the higher PCC value.

### PMA increased calcineurin/calmodulin A interaction and ser9 phosphorylation of GSK3β

**Fig. 3:**
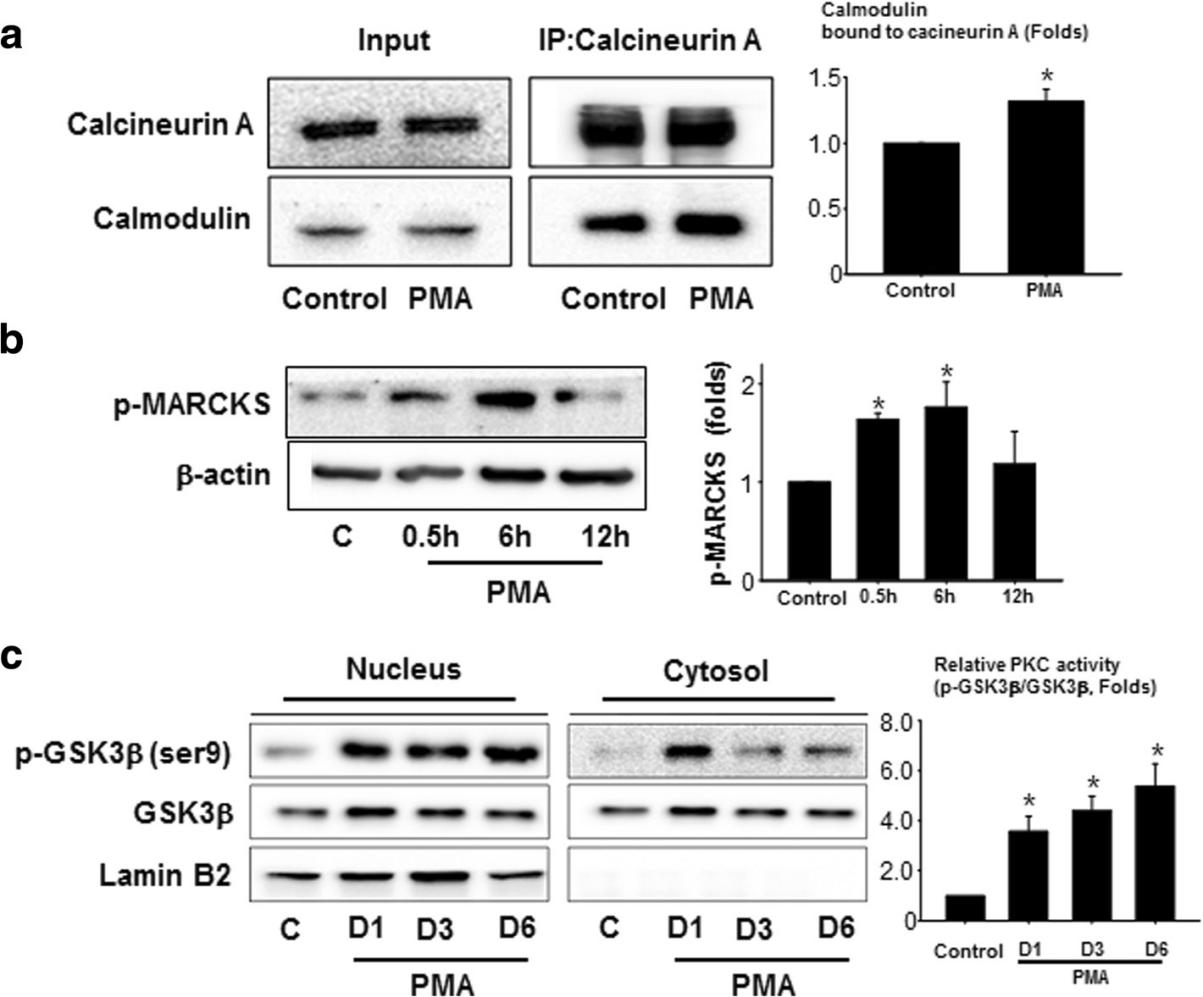
PMA increases calmodulin-calcineurin interactions and phosphorylation of GSK3β. **a** hMSCs treated with PMA (1 μM) for 12 h were immunoprecipitated with anti-calcineurin A. The cell lysates and immunoprecipitates were analyzed by Western blot using the indicated antibodies. **b** The effect of PMA on phosphorylation of MARCKS. The phosphorylation status of MARCKS for the first 12 h of PMA treatment was examined by Western blot using p-MARCKS specific antibodies. **c** The amount of p-GSK3β in the nuclear/cytoplasmic fraction was examined by Western blotting after PMA (1 μM) treatment. Lamin B2 was used as a loading control for the nuclear fraction. *p < 0.05, versus control. *c* control, *D1* 1 day after PMA treatment, *D3* 3 days after PMA treatment, *D6* 6 days after PMA treatment, *GSK3β* glycogen synthase kinase 3 beta, *IP* immunoprecipitation, *MARCKS* myristoylated alanine-rich C kinase substrate, *PKC* protein kinase C, *PMA* phorbol 12-myristate 13-acetate

The nuclear localization of NFAT was more pronounced than that of MyoD after PMA treatment, and this result may indicate that the effect of PMA was mainly mediated by NFAT. Thus, we further examined the underlying mechanisms of PMA with a focus on NFAT. Dephosphorylation of NFAT by phosphatases, such as calcineurin, and the subsequent nuclear translocation of NFAT increases its transcriptional activity [17]. Because the interaction with calmodulin facilitates the activation of calcineurin [18], we examined the interactions between calmodulin and calcineurin using the IP method. After 12 h of PMA (1 μM) treatment, the interaction between calcineurin A and calmodulin increased compared with that of the control, suggesting that PMA induced calmodulin-dependent activation of calcineurin A (Fig. 3a). To examine whether myristoylated alanine-rich C kinase substrate (MARCKS) contributed to this PMA-induced interaction of calcineurin and calmodulin, we checked the effect of PMA on MARCKS phosphorylation. PMA increased phosphorylation of MARCKS up to the first 6 h and then the phosphorylation returned to baseline after 12 h (Fig. 3b), demonstrating that PMA-induced phosphorylation of MARCKS may have contributed to the increased calcineurin-calmodulin interaction. Another possible mechanism may involve inactivation of glycogen synthase kinase 3 beta (GSK3β), which facilitates the phosphorylation of NFAT and subsequent nuclear exit [19, 20]. According to our data, PMA increased the phosphorylation of GSK3β at serine 9, which indicated greater inactivation of GSK3β [21] compared with that of the untreated control (Fig. 3c). Although the amount of phosphorylated GSK3β in the cytoplasmic fraction peaked at day 1 and decreased thereafter, it was still pronounced until day 6 compared with the control.

### PMA induced the transcriptional activity of NFAT

We constructed a GFP vector containing the NFAT binding motif of human interleukin-2 promoter (pAc-NFAT-GFP; Additional file 1: Figure S1A). When pAc-NFAT-GFP (500 ng/well, six-well plate)-transfected Hela cells were treated with 1 μM PMA for 24 h, the expression of GFP was pronounced compared with that of vehicle (DMSO)-treated pAc-NFAT-GFP-transfected cells (Additional file 1: Figure S1B). This result suggested that PMA induced the nuclear translocation and subsequent transcriptional activation of NFAT.

### PMA induced p27 upregulation and proliferation arrest

**Fig. 4:**
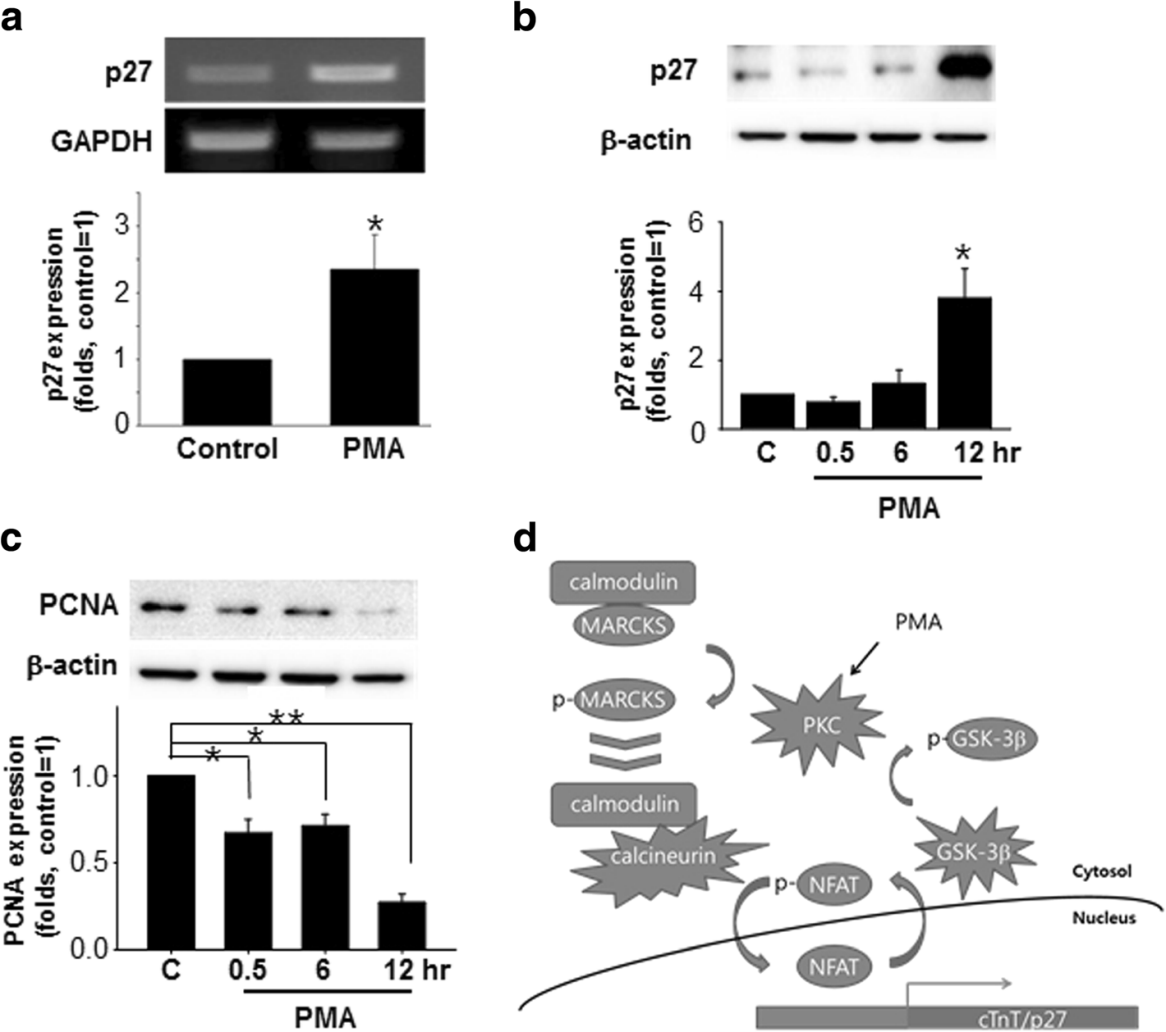
PMA enhances p27 expression in hMSCs. **a** hMSCs were treated with PMA (1 μM) for 12 h. The expression of p27^kip1^ (*p27*) mRNA was measured by RT-RCR. **b** The p27 protein expression level was examined by Western blotting. The cells were treated with 1 μM PMA for up to 12 h. **c** PCNA expression was detected by Western blotting. **d** Schematic drawings of PMA-activated signaling pathways that lead to NFAT activation. *p < 0.05, **p < 0.01, versus control. *GSK3β* glycogen synthase kinase 3 beta, *MARCKS* myristoylated alanine-rich C kinase substrate, *NFAT* nuclear factor of activated T cells, *PCNA* proliferating cell nuclear antigen, *PKC* protein kinase C, *PMA* phorbol 12-myristate 13-acetate

Temporal antagonism between cell cycle exit and differentiation has been demonstrated in cultured cells and animal models [22]. Discovered as an inhibitor of cyclin E/CDK2 (cyclin-dependent kinase 2) [23], the cyclin-dependent kinase inhibitor 1B (p27^kip1^) is a cell cycle inhibitor protein that stops or slows down the cell division cycle. Because NFAT-mediated regulation of p27^kip1^ has been reported [24, 25], we also examined the effect of PMA on the expression of p27^kip1^ and cellular proliferation. Our data indicated that PMA treatment increased the expression of p27^kip1^ at both the RNA (Fig. 4a) and protein (Fig. 4b) levels. Furthermore, PMA significantly decreased the expression of proliferating cell nuclear antigen (PCNA), suggesting that PMA induced cell cycle arrest in hMSCs (Fig. 4c).

## Discussion

Here we demonstrated that NFAT is a key factor that mediates PMA-induced cardiac differentiation of hMSCs. In resting cells, the NFAT regulatory domain is phosphorylated by different kinases, including GSK3 [26–28]. This phosphorylation of the NFAT is important for the activation of NFAT because the phosphorylation status facilitates NFAT nuclear translocation [29]. Multi-phosphorylation at this domain masks the nuclear localization sequence (NLS) of NFAT, resulting in its cytoplasmic localization [30]. Therefore, either promoting the dephosphorylation of NFAT or suppressing its phosphorylation, or both, can lead to nuclear translocation and subsequent transcriptional activation of NFAT. Our data suggest that PMA is involved in both of these mechanisms.

PMA is an activator of PKC [31], and activated PKC can both stimulate dephosphorylation and inhibit phosphorylation of NFAT. First, the PKC-dependent stimulation of NFAT dephosphorylation can be achieved by regulating calmodulin-calcineurin interactions. MARCKS is a PKC substrate that has a calmodulin-binding domain [32], and it is reportedly expressed in MSCs [33]. Upon phosphorylation by PKC, MARCKS dissociates from calmodulin [34]. This result suggests that phosphorylation of MARCKS by PKC can lead to a significant increase in the local concentration of ‘free’ calmodulin that can bind to calcineurin (Fig. 4d). Because binding calmodulin allows calcineurin to exert its phosphatase activity [18], the activation of PKC by PMA may lead to the dephosphorylation of NFAT via the phosphorylation of MARCKS and a subsequent calmodulin/calcineurin interaction. In fact, our data indicated that PMA induced phosphorylation of MARCKS supporting MARCKS-mediated calcineurin/calmodulin interaction.

On the other hand, the activation of PKC may result in the suppression of NFAT phosphorylation by modulating GSK3β activity. Our data clearly demonstrated that PMA induced the phosphorylation of GSK3β (Fig. 3b), which is most likely mediated by PMA-activated PKC [35]. One thing should be noted: there was a transient upregulation of GSK3β after PMA treatment, with unknown mechanisms. Even after an extensive literature search the exact mechanism could not be found. However, since the activity of GSK3β is mainly regulated by its phosphorylation rather than expression itself, as long as PMA treatment increased the phosphorylation of GSK3β, transient upregulation of GSK3β would not have significantly affected the result of this study.

Because the phosphorylation of GSK3β at Ser9 inhibits the kinase activity of GSK3β [21], the PMA-induced phosphorylation of GSK3β may decrease the possibility for NFAT phosphorylation by GSK3β. In turn, the decreased phosphorylation of NFAT results in the increased transcriptional activity of NFAT (Fig. 4d). Although it is difficult to state that NFAT is the single most important transcription factor driving PMA-mediated cardiac differentiation of MSCs, our study provides sufficient evidence that PMA activates the transcriptional activity of NFAT and a logical explanation for how PMA treatment leads to the transcriptional activation of NFAT. Although the GSK3β pathway and calmodulin pathway are discussed separately, there is also high possibility that those two pathways interact at some point to concert the effect of PMA. As far as we know, there is no previous study that has either proved or disproved GSK3β and calmodulin (or calcineurin) interaction. Therefore, it would make a very interesting subject for future study.

Small molecules have great potential in controlling stem cell fate by regulating cellular processes such as proliferation, reprogramming and differentiation [36, 37]. In fact, a number of small molecules have been utilized to differentiate stem cells into cardiomyocytes [4–8], and it is expected that more small molecules will be tested for their abilities to regulate stem cell differentiation. Therefore, it is important to understand how small molecules induce stem cell differentiation because elucidating the underlying mechanism enables us to develop a strategy for synthesizing novel and improved differentiation-inducing small molecules. From that point of view, our study provides valuable information on the cardiac differentiation ability of PMA.

## Conclusion

Our data demonstrated that the PKC activator PMA induced transcriptional activity of NFAT via both GSK3β-mediated pathway and calcineurin/calmodulin-mediated pathway, leading to cardiac-specific marker expression. The results of this study recapitulate the importance of transcriptional regulation during MSC differentiation and help us to better understand the underlying mechanisms of small molecule-mediated MSC differentiation.

## Abbreviations

BMP, bone morphogenetic protein; CDK, cyclin-dependent kinase; cTnT, cardiac troponin T; GFP, green fluorescent protein; GSK3β, glycogen synthase kinase 3 beta; hMSC, human mesenchymal stem cell; IP, immunoprecipitation; MAPK, mitogen-activated protein kinase; MARCKS, myristoylated alanine-rich C kinase substrate; MHC, myosin heavy chain; MSC, mesenchymal stem cell; MyoD, myogenic differentiation 1; NFAT, nuclear factor of activated T cells; PCC, Pearson’s correlation coefficient; PKC, protein kinase C; PMA, phorbol 12-myristate 13-acetate; RT-PCR, reverse transcription polymerase chain reaction

## Additional file

Additional file 1: PMA increases the transcriptional activity of NFAT. (PDF 168 kb)
