# Supplementary material for: The role of nuclear factor of activated T cells during phorbol myristate acetate-induced cardiac differentiation of mesenchymal stem cells
Source: Stem Cell Res Ther. 2016 Jul 12;7:90. doi: 10.1186/s13287-016-0348-6 (PMC4942985; doi:10.1186/s13287-016-0348-6)
Supplement: Additional file 1: — PMA increases the transcriptional activity of NFAT. (PDF 168 kb) [file 13287_2016_348_MOESM1_ESM.pdf]

Supplementary Materials

The role of nuclear factor of activated T cells during phorbol myristate acetate-induced cardiac differentiation of mesenchymal stem cells

Hyang-Hee Seo, Chang Youn Lee, Jiyun Lee, Soyeon Lim, Eunhyun Choi, Jong-Chul Park, Seahyoung Lee, Ki-Chul Hwang

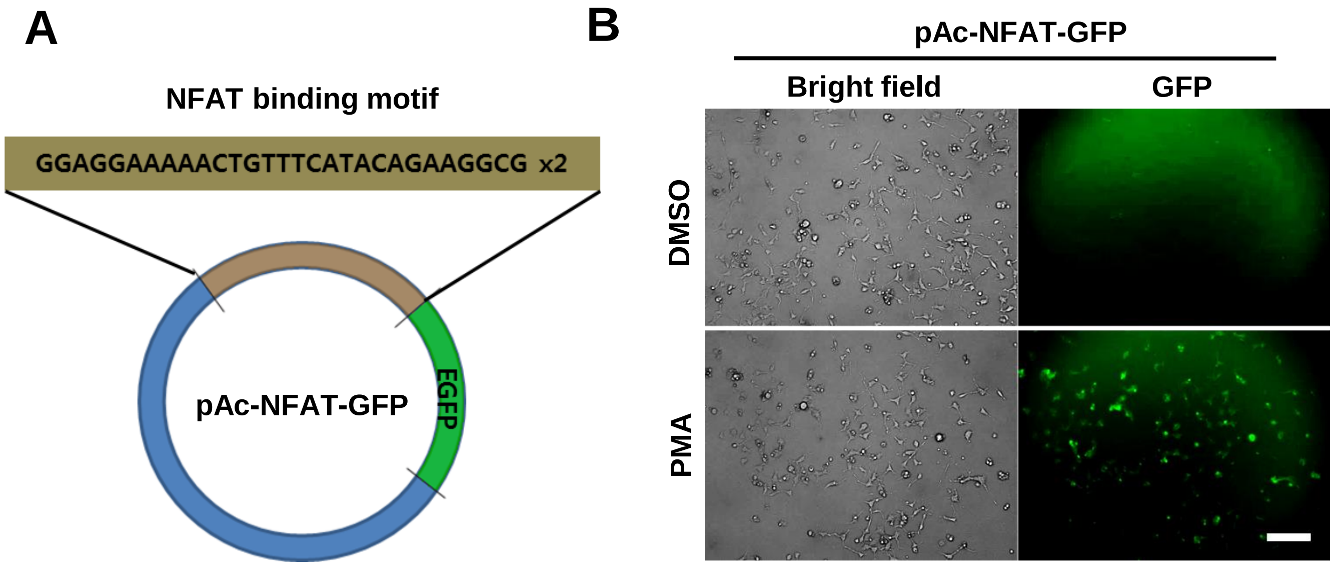

Supplementary Figure S1. PMA increases the transcriptional activity of NFAT. (A) Schematic drawing of the NFAT-driven GFP vector. The pAc-NFAT-GFP vector contained the NFAT-binding motif found in the human interleukin-2 promoter. (B) HeLa cells were transfected with pAc-NFAT-GFP and then treated with PMA (1 μM) for 24 hr. Scale bar=200 μm..
